# Supplementary material for: Immune and non-immune cell fencing of tumor cells is a widespread and functionally relevant spatial pattern in solid cancers
Source: Comput Struct Biotechnol J. 2025 Nov 28;27:5537–48. doi: 10.1016/j.csbj.2025.11.062 (PMC12722985; doi:10.1016/j.csbj.2025.11.062)
Supplement: Supplementary file 1 — Supplementary material [file mmc1.docx]

**Supplementary Information for “Immune and non-immune cell fencing of tumor cells is a widespread and functionally relevant spatial pattern in solid cancers”**

**Table S1:**

 A table providing the cell phenotypes in the order (left to right) in which their fencing slide percentage appears in **Figure 1e**. For each cancer type-cell phenotype bin, the corresponding list of cell sub-phenotypes are listed in order of appearance from left to right in **Figure 1e**. The number of patient slides in which the fencing of the corresponding cell type was evaluated and the percentage which do display fencing significantly (p<0.05) is provided. Each cell phenotype given was defined by the authors of the corresponding studies.

**Table S2:**

**GZMB + CD8+ T cells**

| **Parameter,** $\boldsymbol{i}$ | **Initial Parameter Value** | **Parameter change,** $\boldsymbol{\delta}$ | $\boldsymbol{f}_{\boldsymbol{i,-\delta}}$ | $\boldsymbol{f}_{\boldsymbol{i,+\delta}}$ |
| --- | --- | --- | --- | --- |
| **Contact radius** | $r_{c}=30 \mu m$ | $5 \mu m$ | -0.398 | 0.174 |
| **Cells in a cluster** | $N_{F}=3 cells$ | $1 cell$ | -0.260 | 0.070 |

**TCF1+ CD4+ T cells**

| **Parameter** | **Initial Parameter Value** | **Parameter change,** $\boldsymbol{\delta}$ | $\boldsymbol{f}_{\boldsymbol{i,-\delta}}$ | $\boldsymbol{f}_{\boldsymbol{i,+\delta}}$ |
| --- | --- | --- | --- | --- |
| **Contact radius** | $r_{c}=30 \mu m$ | $5 \mu m$ | -0.256 | -0.237 |
| **Cells in a cluster** | $N_{F}=3 cells$ | $1 cell$ | -0.161 | -0.024 |

**FPM sensitivity analysis.** The FPM is dependent on two parameters external to the data: the radius of contact between two cells, $r_{c}$, and the minimum number of cells which constitute a fencing cluster, $N_{F}$. Given the distribution of FPMs over all patient slides, we then average the FPMs over groups of patient slides to compare their averages between patients with different clinical outcomes. We ultimately compare the average FPMs to discover which patient group has a higher average FPM. To find how the relationships between the average FPMs deviate with variations in these parameters, we consider the patient groups of non-responders and responders to therapy in the TNBC dataset. We first compute the average FPMs with the parameters used in the study for both responders, $\left\langle FPM \right\rangle_{R,b}$, and non-responders, $\left\langle FPM \right\rangle_{NR,b}$, to therapy, and find their difference, $d_{b}=\left\langle FPM \right\rangle_{R,b}-\left\langle FPM \right\rangle_{NR,b}$. We then perturb $r_{c}$ or $N_{F}$ independently, denoted by from $i$, by adding a factor $\pm\delta$, and calculate, $d_{i,\pm\delta}=\left\langle FPM \right\rangle_{R,i,\pm\delta}-\left\langle FPM \right\rangle_{NR,i,\pm\delta}$, as well. Finally, we find the fraction of $d_{b}$ by which $d_{\pm\delta}$ varies from $d_{b}$, $f_{i,\pm\delta}=\frac{d_{i,\pm\delta}-d_{b}}{d_{b}}$. This metric is 0 when $d_{b}=d_{i,\pm\delta}$. We perform these calculations with two different cell types, GZMB + CD8+ T cells and TCF1+ CD4+ T cells, to survey the effects of these perturbations of the FPM parameters across cell types. The base average FPM differences for these two cell types are -0.077 and 0.153 respectively.

**Figure S1. Fencing participation metric for different cell types is associated with survival of patients in HNC.** For HNC, we find that the survival of patients with high (above average) and low (below average) fencing participation metric calculated for certain cell types differ significantly (using the log-rank test, p<0.05). Here we show the survival curves for those cell types. Each curve is generated by the survival data yielded from over 100 patients.

**Figure S2. Tumor access ratio with “hairy” fence geometry. (a)** Plot of tumor access ratio for different hairy fence lengths at various times. Dots correspond to tumor access ratio, $R_{b}\left( T \right)$, calculated from simulations of the hairy fence model. As before, the ratio decreases from 1 (full access) with no fence to 0 (no access) when the fence covers the entire tumor boundary. The tumor access ratio is calculated here identically as before except the initial conditions are shifted up away from the fence by one pixel to leave space for the hairy fence cells. We see that the tumor access ratio has a similar relationship with fence length and simulation time in the context of a hairy fence as it does in the context of a linear fence. **(b)** The difference in the tumor access ratios between the hairy fences and the linear fences for multiple length and time scales. We see that the tumor access ratio in the system with the hairy fences is generally reduced and is often most reduced for longer time scales. Ultimately, there tumor access ratios only differ by ~0.01 (only 1 cell out of 100) which is a small difference. The tumor access ratios for the hairy and linear fences are calculated using the same initial conditions as outlined in (a).

**Figure S3. Characterizing the relation between cell density and the fencing participation metric for TNBC.** The correlation of $c(r)=(1-r)\frac{\sigma_{A}}{\sigma_{max}}-rM$ with patient response in TNBC plotted against the ratio r for **(a)** GZMB+ CD8+ T cells and **(b)** CD4+ PD1+ T cells. At r=0, we find the correlation of cell density with response. In **(a),** introducing the contribution of the fencing participation metric to $c(r)$ by increasing r, increases the correlation with response before it decreases. In **(b)**, the fencing participation metric has a stronger correlation with response than the density for CD4+ PD1+ T cells.

**Figure S4. Characterizing the relation between FPM and the Ripley’s K cross function in TNBC.** The correlation of $f_{R}\left( r_{c};\omega\right)=\omega\frac{R_{k}\left( CD8,CD8;r_{c} \right)}{R_{k,max}\left( CD8,CD8;r_{c} \right)}+\left( 1-\omega\right)\frac{R_{k}\left( CD8,Cancer;r_{c} \right)}{R_{k,max}\left( CD8,Cancer;r_{c} \right)}$ with the GZMB+ CD8+ T cell FPM in TNBC plotted against the weight $\omega$. At $\omega=0$, we find the correlation of the FPM with Ripley’s K cross function of GZMB+ CD8+ T cells with cancer cells whereas at $\omega=1$, there is the correlation of the FPM with Ripley’s K cross function of GZMB+ CD8+ T cells with GZMB+ CD8+ T cells. The FPM has a maximum correlation with the metric at $\omega=0.4.$
